# Exercise Volume Can Modulate the Regenerative Response to Spinal Cord Injury in Mice

**DOI:** 10.1089/neur.2024.0023

**Published:** 2024-07-31

**Authors:** Anne Caroline Rodrigues dos Santos, Renata Pereira Laurindo, Fernanda Marques Pestana, Luiza dos Santos Heringer, Nathalie Henrique Silva Canedo, Ana Maria Blanco Martinez, Suelen Adriani Marques

**Affiliations:** ^1^ Graduate Program in Pathological Anatomy, Federal University of Rio de Janeiro, Rio de Janeiro, Brazil.; ^2^ Neurobiology Department, Institute of Biology, Federal Fluminense University, Rio de Janeiro, Brazil.

**Keywords:** exercise, functional recovery, morphological assessment, nerve regeneration, spinal cord injury

## Abstract

Traumatic spinal cord injury (SCI) causes debilitating motor and sensory deficits that impair functional performance, and physical rehabilitation is currently the only established therapeutic reality in the clinical setting. In this study, we aimed to assess the effect of exercise of different volume and timing of intervention on functional recovery and neuromuscular regeneration in a mouse model of compressive SCI. Mice were assigned to one of four groups: laminectomy only (SHAM); injured, without treadmill training (SCI); injured, treadmill trained for 10 min until day 56 postinjury (TMT1); and injured, treadmill trained for two 10-min cycles with a 10-min pause between them until day 28 postinjury followed by the TMT1 protocol until day 56 postinjury (TMT3). On day 7 postinjury, animals started an eight-week treadmill-training exercise protocol and were trained three times a week. TMT3 mice had the best results in terms of neuroregeneration, functional recovery, and muscle plasticity as measured by functional and morphometric parameters. In conclusion, the volume of exercise can modulate the quality of the regenerative response to injury, when started in the acute phase and adjusted according to the inflammatory window.

## Introduction

Spinal cord injury (SCI) is a debilitating condition that triggers physical, social, and emotional disorders.^1–3^ There are approximately 2.5 million people living with SCI worldwide, with an estimated 170,000 new cases each year.^4^ Functional deficits following SCI are caused by a primary mechanical injury followed by a secondary cascade of events triggered by Wallerian degeneration, excitotoxicity, inflammation, oxidative stress, and blood–spinal cord barrier alterations.^5–7^

The level and severity of the lesion affect an animal’s morbidity and mortality rates after SCI.^8^ In the current study, we used a mouse thoracic SCI model,^8^ which is similar to human lesions because it results from a combination of contusion and compression.^9–12^ Also, this SCI model preserves the lumbar motor neurons and the circuitry involved in the automatic gait pattern (i.e., the central pattern generator, CPG) required for locomotion.^11^

Treadmill exercise is the most common experimental paradigm to study the adaptations induced by exercise and allows the investigator to control the mice workload through the training easily modifiable parameter selection and quantification. American Physiological Society defines three different training intensity levels as follows: low, moderate, or high intensity, and the effects of exercise at each intensity level result in a different training effect.^13^ As the sex and the strain of mice can influence the variability of training performance, researchers commonly conduct an exhaustion test to determine each animal’s exercise capacity before training. The acceptable outcome measure of training efficacy included assessment of maximal speed, maximal oxygen (VO_2_ max) uptake, maximal blood lactate steady state, and maximal heart rate. Heart rate and VO_2_ max increase linearly as a function of either duration or intensity of work.^13^ However, it seems complicated to assess these parameters when the training occurs after surgery or injury that compromises the locomotor performance. Therefore, most exercise studies in SCI still use fixed speed as the training standard.

Treadmill training leads to direct adaptations in the skeletal muscle systems and a systemic effect on the body due to the biochemical, molecular, genetic, and epigenetic mechanisms that underlie the body’s adaptive responses to physiological stress.^11,14^ Regular exercise shows these favorable effects mediated, in part, by the release of circulating bioactive factors during exercise.^14^ Treadmill exercise offers significant physiological adaptations that help in the postinjury recovery process. These exercise-induced adaptations in several parameters contribute to the oxygenation of nervous tissue (such as cardiac output, blood pressure, partial pressure of oxygen (pO_2_), and partial pressure of carbon dioxide (pCO_2_) in blood), balancing secondary postinjury response events, such as local ischemia and lipid peroxidation.^12–14^ Furthermore, regular treadmill exercise improves sensory and motor function in animals and humans,^15^ as well as preventing muscle atrophy and joint contractures, increasing muscle glucose uptake, and preserving muscle mass and motor neuron function.^14,16,17^ Locomotor recovery after complete or severe SCI depends on the presence of CPG, which can generate the complex sequential activation of various leg muscles, working in conjunction with sensory feedback from the legs.^11,15^ Locomotor training after SCI is based on the principle that sensory inputs reactivate and reorganize the spinal locomotor circuitry by exerting an excitatory effect on extensor muscles, thus increasing the activity of these muscles during the stance phase.^11,18,19^

Exercise is also capable of generating endogenous neuroprotection through the activation of multiple mechanisms, including the promotion of neurogenesis, improvement of neurovascular unit integrity, reduction of apoptosis, rescuing of synapse plasticity, and modulation of neuroinflammation.^20^ These beneficial effects may be attributed to increased levels of neurotrophins such as brain-derived neurotrophic factor (BDNF), neurotrophin-3 (NT-3), and neurotrophin-4 (NT-4) in the spinal cord and skeletal muscles of exercised animals.^14,20^ Treadmill training is generally chosen due to the high response of aerobic metabolism to training.^21–25^

The effectiveness of physical training after SCI is dependent on lesion severity, onset of training, and specificity of the lost activity originated by the injury.^26^ The mechanisms by which physical rehabilitation improves function after injury in humans need to be better understood so that exercise can be adequately controlled and exploited therapeutically. Thus, standardized trials in experimental lesion models are extremely important to test the effectiveness of different exercise paradigms such as the type, volume, frequency, and timing of the intervention.^27^ All these parameters can positively influence the regenerative and functional results; however, the literature also shows negative results associated with exercise volume and load or early onset after injury.^28–31^ Treadmill training has demonstrated good results, depending on the protocol, proving to be an important therapy, with good potential for applicability.^30,31^

This study aimed to assess the morphofunctional and immunomodulatory effects of different treadmill training volumes, defined according to the postinjury time of the SCI (subacute or chronic) in which the training was performed.^12,13^ Our results showed that the exercise-trained animals exhibited better locomotor performance and tissue preservation associated with a reduction of muscle atrophy when compared with untrained animals. In addition, better results in terms of morphofunctional outcomes were obtained when we adjusted the volume of the treadmill training protocol according to the SCI phase.

## Materials and Methods

The experimental procedures were approved by the Federal University of Rio de Janeiro Animal Care Committee (CEUA/UFRJ, protocol number 01200.001568/2013-87).

### Spinal cord surgery

Eight-to-ten-week-old female mice were anesthetized intraperitoneally with ketamine and xylazine (100 and 15 mg/kg, respectively) and subjected to laminectomy followed by a compressive SCI; the injury was induced with the use of a 30-g vascular clip (Kent Scientific Corporation, Torrington, CT) that was applied for 10 sec at the T9 level. A subcutaneous saline injection (1–2 mL) with antibiotics (Flotril 2.5 mg/kg/d; Bayer, São Paulo, SP, Brazil) was given to the animals immediately after surgery. Injured animals received manual bladder expression twice daily until the return of bladder function.

### Treadmill training

Animals ran on a motor-driven treadmill (Insight, Ribeirão Preto, SP, Brazil) at a speed of 6–12 m/min according to the each animal’s tolerance. The protocol was established in accordance with literature^12^ and data obtained from previous studies by our group.^31^ The effects of the volume of exercise on morphofunctional outcomes after SCI were tested using two different treadmill training protocols TMT1 (injured mice treadmill trained for 10 min until day 56 postinjury) and TMT3 (injured mice treadmill trained for two 10-min cycles with a 10-min pause between them until day 28 postinjury and the TMT1 protocol until day 56 postinjury). The training was performed three days per week, starting on day seven after injury up to eight weeks. The trained groups’ (TMT1 and TMT3) outcomes were compared with the injured (SCI) and not injured (SHAM) untrained groups to assess protocol training effects.

### Behavioral analysis

The Basso mouse scale (BMS),^32^ the rotarod test,^33^ and the ladder walking test (LWT)^34^ were used to assess the animals’ motor behavior. For the BMS, the score for each hindlimb was recorded as an average per animal (left and right hindlimb) to obtain one BMS score per mouse, and then the mean of the group was calculated (*n* = 6 for each group). The BMS was performed before surgery and then weekly up to eight weeks after surgery.

The rotarod was used to evaluate the influence of exercise on mouse motor coordination and functional performance of the animal after SCI. Therefore, the number of falls from the rotarod made by each mouse 3 min^33^ at a fixed speed of 25 rpm was recorded, to set a default speed. This test was performed at 28, 42, and 56 days after SCI (*n* = 6 for each group).

The LWT was performed weekly from day 28 until day 56 postinjury. Animals were required to cross a horizontal ladder on which the spacing of the rungs was variable and changed periodically to minimize the ability of the animals to compensate the impairments through learning. A webcam (Microsoft, Redmond, WA) was used to record the positions of the limbs for each animal (30 f/sec, three records for each side). The number of total steps was recorded for each animal (*n* = 6 for each group).

### Mechanical sensitivity assessment

The mechanical sensitivity was assessed using an electronic von Frey apparatus (digital analgesiometer, Insight) to measure the mouse hind paw withdrawal threshold (PWT). Mice were acclimated for 15 min in the appropriate chambers before PWT assessment. A polypropylene tip was used in the probe cone of the transducer^35^ and the maximum pressure applied was recorded on the display screen when the animal removed the paw.

### Electroneuromyography analysis

Electroneuromyography (ENMG) analysis was performed at the last day of survival using a PowerLab^®^ 4/35 system (AD Instruments, Sydney, Australia) and LabChart application program. Laminectomy was performed under anesthesia at the C0-C1 level and the area was stimulated with the stimulation electrode (10 mV stimulus). The active recording electrode was positioned on the gastrocnemius muscle and the compound muscle action potential (CMAP) was evoked a few milliseconds after the stimulus. The neutral electrode was positioned in the dorsal subcutaneous tissue of the animal. Up to five pulses of electric stimuli (10 mV) were applied with a regulated range. For this test, we created the immediately postinjury group (IPI) to evaluate the immediate (10 min after injury) ENMG response pattern.

### White matter sparing

Following intracardiac perfusion, spinal cords (4-mm segments, containing rostral, caudal, and lesion epicenter areas) were harvested, cryoprotected in sucrose-PBS (phosphate-buffered saline) solution, embedded in Tissue-Tek^®^ OCT^IM^ compound (Sakura Finetek, Torrance, CA), and frozen. Ten-micrometer serial cross sections were cut with a CM 1850 cryostat (Leica, Wetzlar, Germany; *n* = 6 per group) and collected in six parallel series of eight slides each. The first slide of each series containing six sections spaced 100 μm apart^9^ was stained with Luxol^®^ fast blue (LFB) stain. Sections were observed under an Axioskop 2 Plus microscope (Zeiss, Oberkochen, Germany) and photographed with an AxioCam MR camera (Zeiss) using AxioVision software version 4.5 (Zeiss) for image acquisition. Quantification was performed using Image JAVA software (ImageJ, Jandel Scientific, Corte Madera, CA) and the spared white matter (total cross-sectional area minus LFB nonstained area) was plotted as a percentage of total area. The results were plotted every 250 μm (0.25 mm), rostrally and caudally to the lesion epicenter.

### Tissue processing for morphometric analysis

Eight weeks after surgery, a spinal cord segment (1 mm long, containing the lesion epicenter) and soleus and gastrocnemius muscles fixed as previously described were dissected and postfixed (1% osmium tetroxide plus 0.8% potassium ferrocyanide in 0.1M cacodylate buffer for 6 h). The spinal cords were dehydrated in a graded acetone series (30, 50, 70, 80, 90, and 100% for 10 min each), embedded overnight in Poly/Bed^®^ 812 resin (EMS, Houston, TX), and polymerized for 48 h at 60°C. Soleus muscles were dehydrated in a graded acetone series (30, 50, 70, 80, and 90% for 20 min each) and infiltrated for 15 days in a resin/acetone (50%/50%) solution and 3 days in pure resin before polymerization for 48 h at 60°C. Semithin sections were cut on an ultramicrotome (RMC Boeckeler, Tucson, AZ), collected on glass slides, and stained with toluidine blue. An Axioskop 2 Plus microscope (Zeiss) equipped with AxioVision version 4.5 (Zeiss) was used for imaging.

### Nerve fiber quantification and g-ratio analysis

The number of preserved spinal cord myelinated nerve fibers was quantified in semithin sections taken from the lesion epicenter (*n* = 6/group) as described by Massoto and collaborators (2020). Digital images were taken in a systematic way at 1000× magnification. Six image fields per animal (three on each side of the spinal cord) were taken in the anterior and lateral white matter areas as illustrated in Figure 4J. ImageJ software (Jandel Scientific) was used to quantify the number of normal myelinated nerve fibers, to determine myelin, axon, and nerve fiber area and to calculate the g-ratio (*n* = 3 each). The g-ratio was calculated as the ratio of the inner axonal diameter and the outer fiber diameter.

### Soleus and gastrocnemius muscle analysis

The soleus and gastrocnemius muscles were dissected and weighted on a precision scale^2^ immediately after perfusion. Images were taken at 200× magnification using an Axioskop 2 Plus microscope (Zeiss) equipped with AxioVision version 4.5 (Zeiss). Total cross-sectional area for each muscle sample was determined with ImageJ software (Jandel Scientific, Corte Madera, CA).

### Muscle histochemistry for ATPases

Fresh gastrocnemius muscles were removed, frozen in liquid nitrogen, and cut (8 μm) with a cryostat (Leica, Teaneck, NJ) for histoenzymatic analysis. We used the alkaline and acid ATPase techniques^36^ to determine fiber type composition. At alkaline pH (9.4), light and dark fibers were identified as slow type I and fast type II, respectively. The acid ATPase reaction (pH 4.3) was used to confirm the results at pH 9.4, because the staining of fiber types is opposite to that of the alkaline reaction. Photomicrographs of each muscle section were taken using an AxioCam MR digital camera (Zeiss) and the total number of fibers was each fiber type. The proportion of each fiber type was expressed as a percentage of the total number of fibers.

### Neurotrophin immunohistochemistry

At 56 days postinjury, spinal cord tissue sections were processed for neurotrophin immunohistochemistry. We used immunoperoxidase techniques with 3,3’-diaminobenzidine (DAB) as a chromogen (Reveal polyvalent HRP–DAB detection system; Spring Bioscience, Pleasanton, CA) to identify the following proteins: NT-4, NT-3, BDNF, and nerve growth factor (NGF) (all antibodies were from PeproTech, Cranbury, NJ). Sections were blocked with normal goat serum (NGS, 10%) and incubated overnight with the primary antibodies (1:100 dilution) in 0.3% Triton X-100 in PBS. Next, sections were washed with PBS, incubated for 2 h with the HRP-conjugate, revealed with DAB, and mounted with fluoromount (Sigma-Aldrich, St. Louis, MO). Images were captured (20× magnification) with an Axioskop 2 Plus microscope (Zeiss) and analyzed using FIJI software (NIH, Bethesda, MD). Twenty-four sections were taken per animal (*n* = 4) 1 mm rostrally to 1 mm caudally to the lesion epicenter. The integrated density of immunoreactive sites was determined as the ratio of the area of interest and the total area of the photographed field.

### Immunofluorescence labeling

Sections were washed three times in 0.1 M blocked PBS (pH 7.4) containing 0.3% Triton X-100 and 2.5% NGS, at room temperature for 1 h, and incubated overnight in one or more of the following antibodies: mouse monoclonal anti-CC1 (anti-adenomatous polyposis coli clone CC1, 1:200, AB16794), rabbit monoclonal anti-GAP-43 (growth-associated protein-43, 1:100, ab75810), rabbit polyclonal anti-GFAP (glial fibrillary acidic protein, 1:100, Sigma-Aldrich), rabbit polyclonal anti-NF200 (neurofilament protein 200, 1:500, ABN76), and rabbit monoclonal anti-TMEM119 (transmembrane protein 119, 1:100, Abcam, Cambridge, UK). Sections were washed three times in PBS, incubated in the appropriate secondary antibodies (Alexa-Fluor 546 goat anti-rabbit, Alexa-Fluor 546 goat anti-mouse, Invitrogen, Waltham, MA) for 2 h at room temperature, counterstained with 4’,6’-diamino-2-fenil-indol (DAPI, Molecular Probes, Eugene, OR) for 5 min, and coverslipped with fluoromount (Sigma-Aldrich) for visualization. Negative control sections were processed identically except that the primary antibodies were omitted. Staining was analyzed and documented on an Axioskop 2 Plus laser scanning microscope (Zeiss). Quantification of immunofluorescence data was performed using ImageJ software (Jandel Scientific, Corte Madera, CA) by determining the integrated density for each section.

### Statistical analysis

All analyses were performed using GraphPad Prism 6.0 software (Graph Pad Software, Boston, MA). Data were analyzed using one-way or two-way analysis of variance (ANOVA) and Student’s *t-test* followed by Tukey’s or Bonferroni post tests. Data are expressed as mean ± SEM, and *p* < 0.05 was considered statistically significant.

## Results

### Exercise volume can modulate functional recovery after SCI in mice

**FIG. 1.**
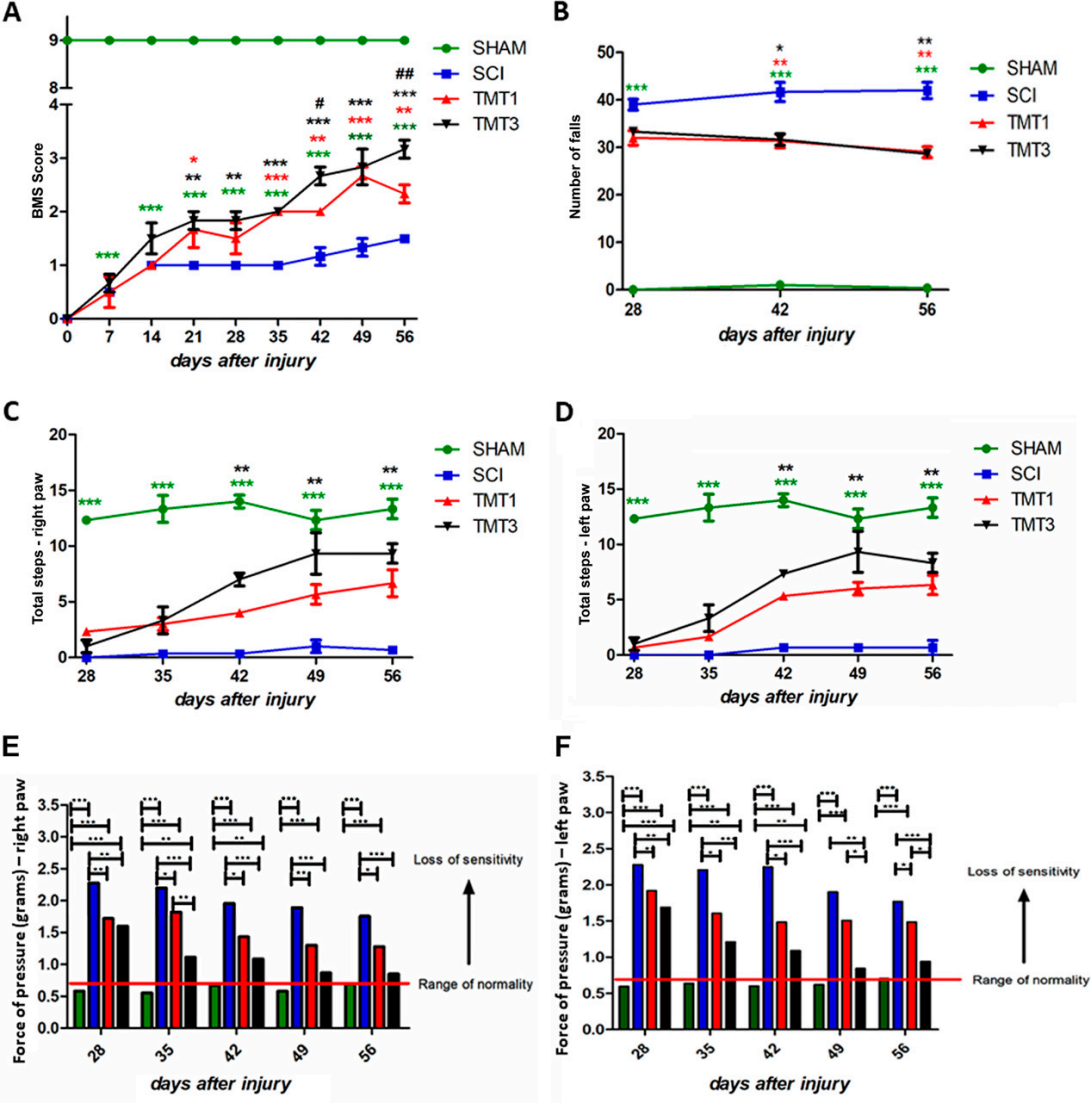
Functional outcomes can improve with exercise depending on the training protocol. **(A)** Evolution of Basso Mouse Scale (BMS) scores for SHAM, spinal cord injury (SCI), TMT1, and TMT3 mice. The exercised groups (TMT1 and TMT3) showed significantly better BMS scores compared with the SCI group. At 42 days after the injury, BMS scores were also better in the TMT3 than in the TMT1 group. **(B)** Comparison of the number of falls in the rotarod test. The exercised groups made a smaller number of falls than SCI mice from 42 days postinjury until the end of the survival period. Data for the ladder walking test showed the total steps from **(C)** right and **(D)** left hind paw for all studied groups. **(E and F)** Von Frey assessment to detected mechanical sensitivity to both hind paws from all studied groups. Data were analyzed using two-way ANOVA and Tukey’s post test.

For all results described below, the SHAM group will be the physiological parameter, with its values being considered to define the limits of normality. The BMS analysis (Fig. 1A) revealed that SHAM animals had normal scores (BMS score 9.00 ± 0.00) throughout the experimental period, whereas injured mice (SCI, TMT1, and TMT3) exhibited hindlimb paralysis (BMS score = 0) immediately postinjury. In addition, the exercised groups (TMT1 and TMT3) showed a significant locomotor improvement 21 days postsurgery (TMT1 2.20 ± 0.29, *p* < 0.05 and TMT3 3.167 ± 0.3402, *p* < 0.01) compared with the SCI group (1.50 ± 0.452). Mice from the TMT3 group showed better results, reaching an intermediate phase of recovery exhibiting plantar placing and the development of stepping, as described by Basso and collaborators (2006), and also had significant scores compared with TMT1 mice (*p* < 0.01) from day 42 postinjury until the end of the trial.

The number of falls from the rotarod made by each mouse over 3 min was recorded (Fig. 1B). SHAM animals (0.333 ± 0.235) maintained their functional performance throughout the experimental period, with few or no falls, and experienced significantly fewer falls than mice in the injured groups (*p* < 0.001). Injured animals exhibited significant functional loss; however, mice in the exercised groups made significantly fewer falls (TMT1 29.0 ± 1.838 and TMT3 28.67 ± 1.472) than SCI mice (40.17 ± 0.986, *p* < 0.01).

At 28 days postsurgery when mice could already walk with plantar foot contact, we counted the number of total steps taken by each mouse in the LWT once weekly until the end of the survival period (Fig. 1C and D). Because of evident asymmetry between the right and left paws, the data were analyzed separately for the right (RP) and left (LP) hind paws. The results showed that TMT3 mice took significantly more steps with both hindlimbs (RP: 9.333 ± 1.663 and LP: 8.333 ± 1.587, *p* < 0.01) than SCI mice (RP: 0.666 ± 0.170 and LP: 1.666 ± 0.163) from day 42 postinjury until the end of the survival period. Although the exercised animals showed better functional recovery, the number of total steps in TMT1 (RP: 6.667 ± 0.987 and LP: 6.333 ± 1.734) was not significantly different from that in SCI. In addition, there was no significant difference in the total number of steps between the exercised groups.

Tactile sensitivity on the plantar surface of the hind paws determined as the force in grams required to elicit a paw withdrawal response was assessed using an electronic von Frey device (digital analgesiometer). We tested both hind paws and data were analyzed separately for the right (RP) and left (LP) paws (Fig. 1E and F). The SHAM values (RP 0.693 ± 0.028 g and LP 0.706 ± 0.023 g) were considered the normal threshold to provoke a paw withdrawal response and were significantly lower compared with the injured groups (*p* < 0.001) throughout the experimental period. Furthermore, the paw withdrawal threshold (PWT) for the exercised groups (TMT1 and TMT3) was significantly lower (TMT3, 28 days: RP 1.60 ± 0.13 g, *p* < 0.01 and LP 1.7 ± 0.22 g, *p* < 0.05; 42 days: RP 1.09 ± 0.13 g and LP 1.27 ± 0.24 g; TMT1, 28 days: RP 1.72 ± 0.11 g, p; <0.05 and LP 1.84 ± 0.20 g; 0.11 g and LP 1.47 ± 0.15 g) compared with the SCI group (2.31 ± 0.19g). The left paw (LP) of TMT3 mice also exhibited significantly better sensory performance than TMT1 mice from day 49 postinjury to the end of the survival period (LP *p* < 0.05).

**FIG. 2.**
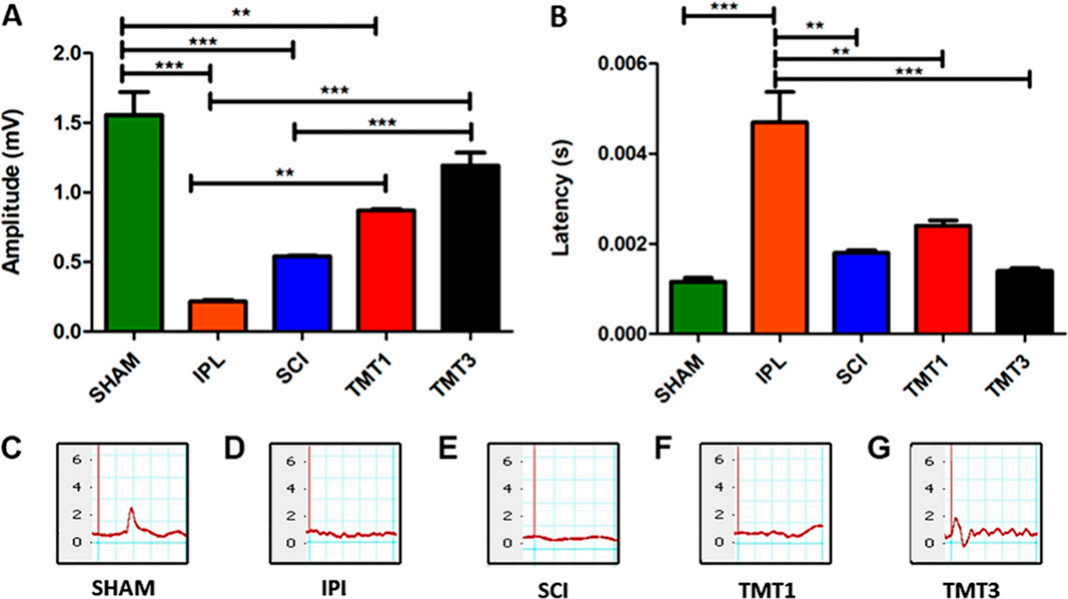
Exercise can improve the amplitude of the compound muscle action potential (CMAP) after compressive spinal cord injury (SCI). The amplitude **(A)** and latency **(B)** of the CMAP were assessed by electroneuromyography (ENMG) in all groups. All injured groups showed a statistically significant decrease in CMAP amplitude and increase in CMAP latency compared with SHAM mice, except for TMT3 mice whose values were closer to normal. CMAP amplitude was also significant in TMT3 mice than in injured groups without training (SCI and immediately postinjury [IPI]) and in TMT1 than in the IPI group. **(C–G)** ENMG traces. **p < 0.01 and ***p < 0.001. Data were analyzed using two-way ANOVA and Tukey’s post test.

We also assessed the amplitude and latency of the CMAP by ENMG (Fig. 2). CMAP amplitude was significantly in the SHAM group (1.61 ± 2.18 mV) when compared with the IPI (0.22 ± 0.74 mV, *p* < 0.001), SCI (0.63 ± 0.318 mV, *p* < 0.001), and TMT1 (0.79 ± 0.54 mV, *p* < 0.01) groups, but not the TMT3 group (1.36 ± 1.92 mV, *p*=ns). Nevertheless, all injured groups showed a reduction in CMAP amplitude (Fig. 2A and C–G) and an increase in CMAP latency (Fig. 2B) relative to SHAM mice. Also, IPI mice showed the lowest CMAP amplitude (0.22 ± 0.74 mV) and highest CMAP latency (0.0046 ± 2.98 sec) of all injured groups (SCI 0.63 ± 0.318 mV and 0.0017 ± 0.87 sec; TMT1 0.79 ± 0.54 mV and 0.0022 ± 1.34 sec; and TMT3 1.36 ± 1.92 mV and 0.0013 ± 0.76 sec; amplitude and latency, respectively). Both parameters were significantly different in the exercised groups (TMT1 *p* < 0.01 and TMT3 *p* < 0.001) compared with the IPI group. In addition, the CMAP amplitude was also significant in the TMT3 group when compared with the SCI group (*p* < 0.001). Lastly, CMAP amplitude and latency for the TMT3 group were very similar to SHAM values.

### Exercise can enhance the preserved white matter after SCI

**FIG. 3.**
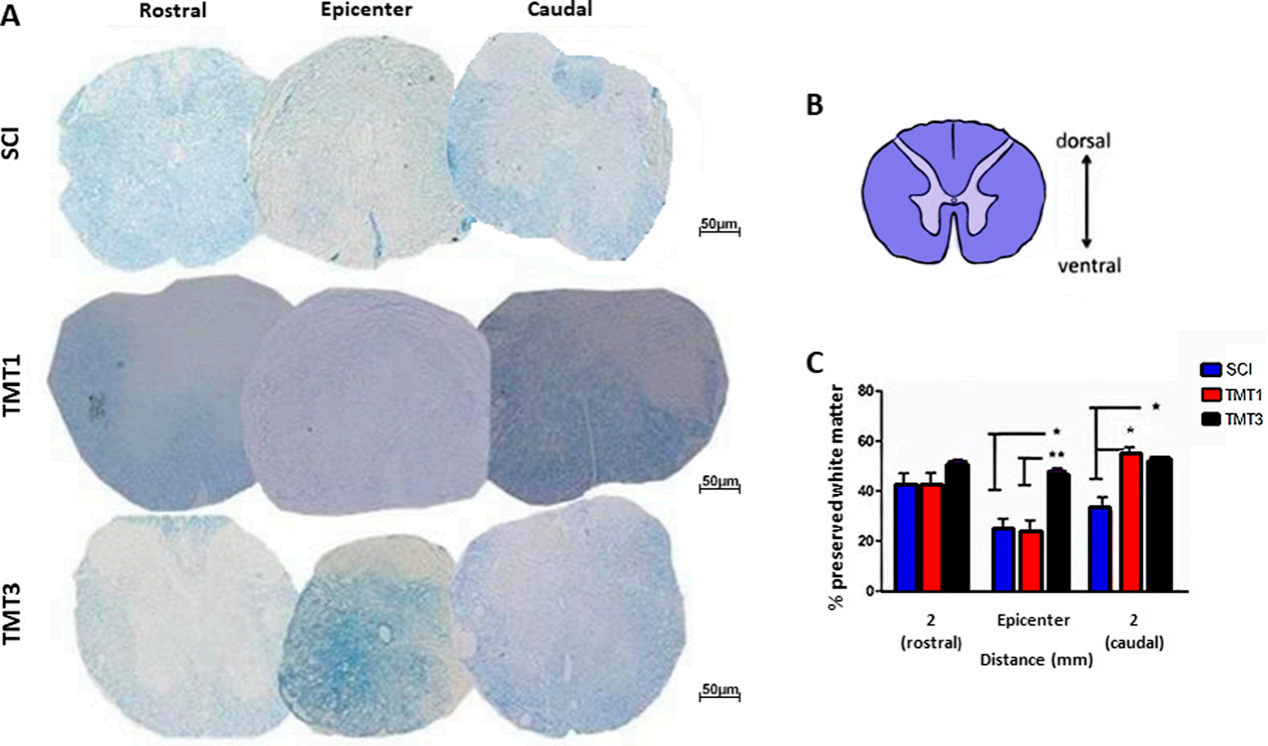
Exercised groups had more preserved white matter after spinal cord injury (SCI). **(A)** LFB-stained spinal cord cross sections at rostral, epicenter, and caudal segments were used to quantify the amount of spared white matter. **(B)** Spinal cord scheme showing the quantified dark blue area. **(C)** Preserved white matter was significantly higher in TMT3 than in SCI and TMT1 at the lesion epicenter, and than in SCI at the caudal area. Also, in TMT1 than in SCI at the caudal area. *p < 0.05 and **p < 0.01. Data were analyzed using one-way ANOVA and Tukey’s post test. Scale bar = 50 μm.

Figure 3 shows spinal cord sections stained with LFB and quantification of the spared white matter. There was no significant difference in spared white matter percent (Fig. 3C) among the injured groups in the rostral area (SCI 43.32 ± 1.91%, TMT1 42.65 ± 1.87%, and TMT3 52.11 ± 0.67%). However, at the epicenter of the lesion of the TMT3 group (48.39 ± 0.675%), percentage of preserved white matter was significantly better than the SCI (25.12 ± 3.763%, *p* < 0.05) and TMT1 (23.57 ± 4.745%, *p* < 0.001) groups. Also, both exercised groups (TMT3 46.98 ± 0.675% and TMT1 49.79 ± 4.745%) showed better white matter preservation in segments caudal to the injury when compared with those of the SCI group (29.32 ± 3.763, *p* < 0.05).

**FIG. 4.**
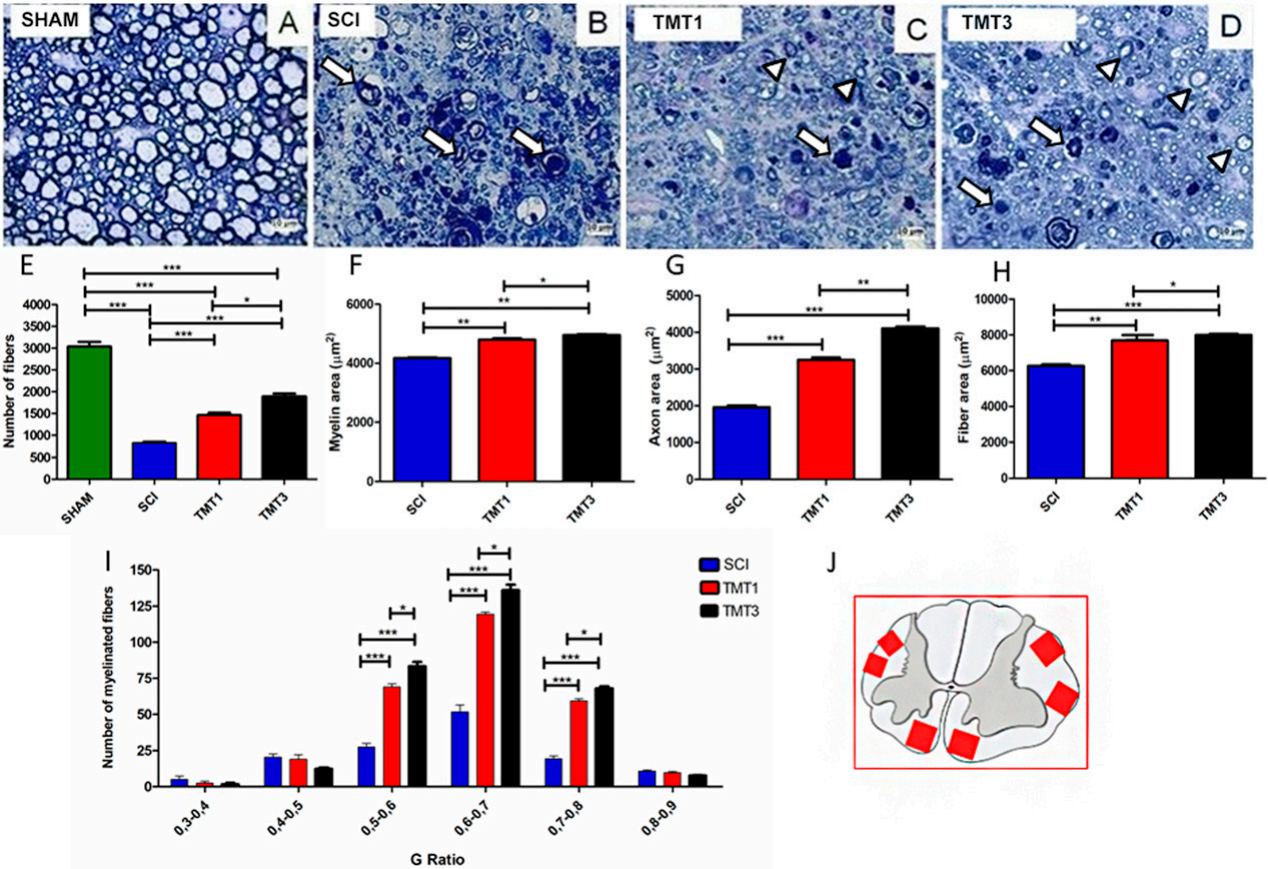
Exercise promotes growth of myelinated nerve fibers with adequate g-ratio values. **(A–D)** Toluidine blue-stained semithin cross sections of the anterolateral funiculus from all groups (arrow: degenerating fibers; arrowhead: regenerating fibers). **(E)** Exercised groups (TMT1 and TMT3) had a larger number of fibers compared with SCI, and TMT3 also had more fibers than TMT1. **(F–H)** Myelin, axon, and fiber areas were significantly larger in the exercised groups than in the SCI, and in TMT3 than in TMT1. **(I)** g-ratio analysis stratified by range. Most nerve fibers from the exercise groups were within the 0.5–0.8 g-ratio range. TMT3 also had more myelinated fibers than SCI and TMT1 across the 0.5–0.8 range. **(J)** Representative scheme of quantified areas. Data were analyzed using one-way ANOVA and Tukey’s post test; *p < 0.05, **p < 0.01, and ***p < 0.001. Scale bar = 20 μm. SCI, spinal cord injury.

Toluidine blue-stained semithin cross sections from day 56 postinjury were used for morphometric analysis. The SHAM group exhibited normal tissue organization (Fig. 4A), whereas the tissue cytoarchitecture was lost in the SCI group (Fig. 4B). The exercised groups (4C: TMT1 and 4D: TMT3) showed better tissue cytoarchitecture than the SCI group but degenerated fibers (arrows) were still seen in the exercised groups. Quantitatively, exercised groups (TMT1 1487 ± 0.26 and TMT3 1982 ± 0.387) exhibited a significant increase in the number of myelinated fibers when compared with the SCI group (64 ± 10.9, *p* < 0.001; Fig. 4E). In addition, the TMT3 group also had a significantly large number of myelinated fibers than the TMT1 ones (*p* < 0.05).

Regarding the myelin and axon area, both exercised groups showed significantly better results when compared with the SCI group (410 ± 0.319µm^2^, *p* < 0.01, Fig. 4F; and 2347 ± 0.4340μm^2^, *p* < 0.01, Fig. 4G). Also, the TMT3 groups (491 ± 0.319µm^2^ and 4896 ± 0.319μm^2^, myelin and axon area, respectively) showed significantly better results than TMT1 (453 ± 0.453µm^2^, *p* < 0.05, and 4126 ± 0.339μm^2^, *p* < 0.01). As a consequence, the myelinated nerve fiber area was significantly better in exercised groups (TMT1 7615 ± 0.478µm^2^, TMT3 7996 ± 0.289µm^2^) than in SCI (SCI 4896 ± 0.319µm^2^, *p* < 0.01 and *p* < 0.001, respectively; Fig. 4H). TMT3 also exhibited a significantly larger myelin nerve fiber area than TMT1 (*p* < 0.05, both).

G-ratio analysis (Fig. 4I) revealed that exercised animals (TMT1 55 ± 2.011 and TMT3 65 ± 1.876) had significantly more fibers with values closer to the optimal range for spinal cord g-ratio (0.7–0.8 range; Chomiak and Hu, 2009) than SCI mice (20 ± 0.923, *p* < 0.001). TMT3 also had significantly more myelinated fibers than TMT1 (*p* < 0.05) across the 0.5–0.8 g-ratio range. Thus, the exercise protocols tested have the potential to promote nerve preservation or plasticity and could ameliorate muscle atrophy.

**FIG. 5.**
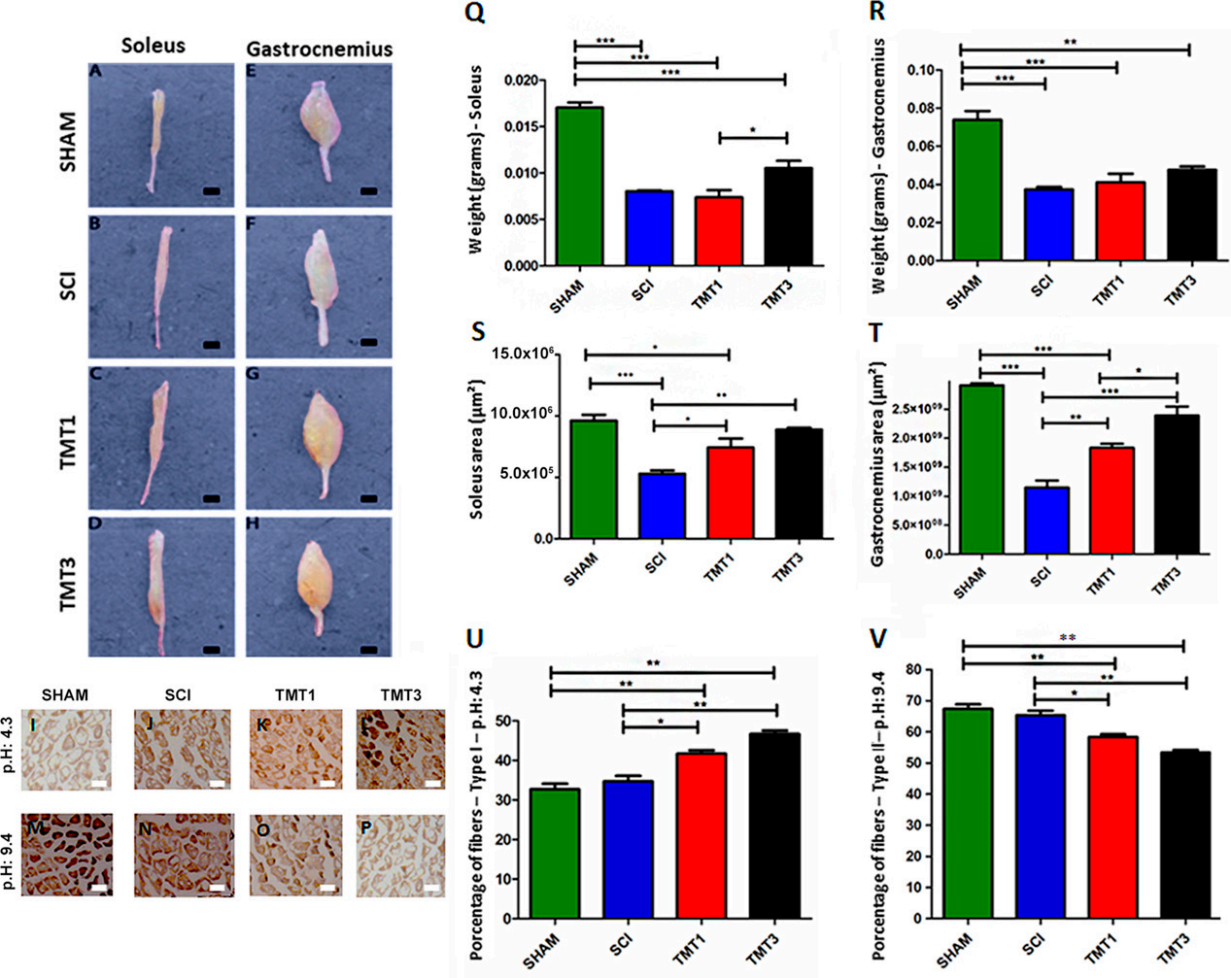
Exercise volume can promote muscle atrophy recovery and muscle plasticity. Samples of soleus **(A–D)** and gastrocnemius **(E–H)** muscles used for weight **(Q–R)** and area **(S–T)** measurements (*n* = 6 each group). The TMT3 exercise protocol had a greater effect on muscle preservation. Photomicrographs of gastrocnemius muscle from acid **(I–L)** and alkaline **(M–P)** ATPase reactions for determination of the type of muscle fiber. **(U–V)** Quantification of type I and type II muscle fibers from acid and alkaline ATPase reaction. SHAM mice showed a predominance of type II fibers in the gastrocnemius, whereas exercised animals showed a predominance of type I fibers, indicating that exercise could stimulate a shift from one type of fiber to another. *p < 0.05, **p < 0.01, and ***p < 0.001. Data were analyzed using one-way ANOVA and Tukey’s post test. Scale bar = 100 μm.

Figure 5 shows the results for soleus and gastrocnemius muscle weight (Fig. 5A–J; *n* = 6 each group). SHAM group weights (soleus 0.0173 ± 0.2476 g and gastrocnemius 0.0723 ± 0.3274 g) were considered the normal pattern and weights for both soleus (SCI 0.0071 ± 0.0035 g, TMT1 0.0068 ± 0.0061 g and TMT3 0.0092 ± 0.0078 g; *p* < 0.001 relative to all injured groups) and gastrocnemius (SCI 0.0373 ± 0.0211 g, *p* < 0.001; TMT1 0.0411 ± 0.0219 g, *p* < 0.001; and TMT3 0.0473 ± 0.0257 g, *p* < 0.01) muscles were significantly lower in the injured groups compared with SHAM weights.

We also determined the cross-sectional area of soleus (Fig. 5K) and gastrocnemius (Fig. 5L) muscles. The soleus area of SHAM (961,337 ± 0.7331µm^2^) was larger than that of injured groups and significantly large compared with SCI (529,795 ± 0.7589µm^2^, *p* < 0.001) and TMT1 (743,668 ± 0.7856µm^2^, *p* < 0.05), but not TMT3 (888,026 ± 0.8646µm^2^). The soleus area was also significantly larger in the exercised groups (TMT1 *p* < 0.05 and TMT3 *p* < 0.01) compared with the SCI group. Notwithstanding the smaller gastrocnemius (TMT1 183,427 ± 1.458 μm^2^ and TMT3 239,001 ± 2.0136 μm^2^) relative to SHAM (291,167 ± 0.5154 μm^2^), the gastrocnemius area in TMT1 and TMT3 was still significantly larger than in SCI (114,825 ± 1.9856µm^2^, *p* < 0.01 and *p* < 0.001, respectively). In addition, the gastrocnemius was significantly larger in TMT3 compared with TMT1 (*p* < 0.05) and in SHAM when compared with SCI and TMT1 (*p* < 0.001). Thus, the exercise protocols tested seem to ameliorate muscle atrophy and promote muscle plasticity.

Histoenzymatic analysis by acid (pH4.3, Fig. 5M–P) and alkaline (pH9.4, Fig. 5Q–T) ATPase was performed to quantify type I (Fig. 5U) and type II (Fig. 5V) muscle fibers from the gastrocnemius, respectively. Acid ATPase enabled identification of type I fibers (i.e., slow fiber), the percentage of which was significantly predominant in the exercised groups (TMT1 39.37 ± 0.756% and TMT3 45.37 ± 0.876%) than in SCI (35.89 ± 1.378%, *p* < 0.05 and *p* < 0.01, respectively) and SHAM (32.75 ± 1.216%, *p* < 0.01). Type II fibers (i.e., fast or intermediate fibers) seen by alkaline ATPase were predominant in the SHAM (77.57 ± 1.308%) and the SCI (75.89 ± 1.348%) groups than in the exercised ones (TMT1 58.11 ± 0.799%, *p* < 0.05, and TMT3 53.97 ± 0.865%, *p* < 0.05 and *p* < 0.01, respectively). SHAM mice showed a predominance of type II fibers in the gastrocnemius, whereas exercised animals showed a predominance of type I fibers, indicating that exercise could stimulate a shift from one type of fiber to another.

### The exercise protocols can influence neurotrophic factor expression, neuroplasticity, glial scar formation, and neuroinflammation

**FIG. 6.**
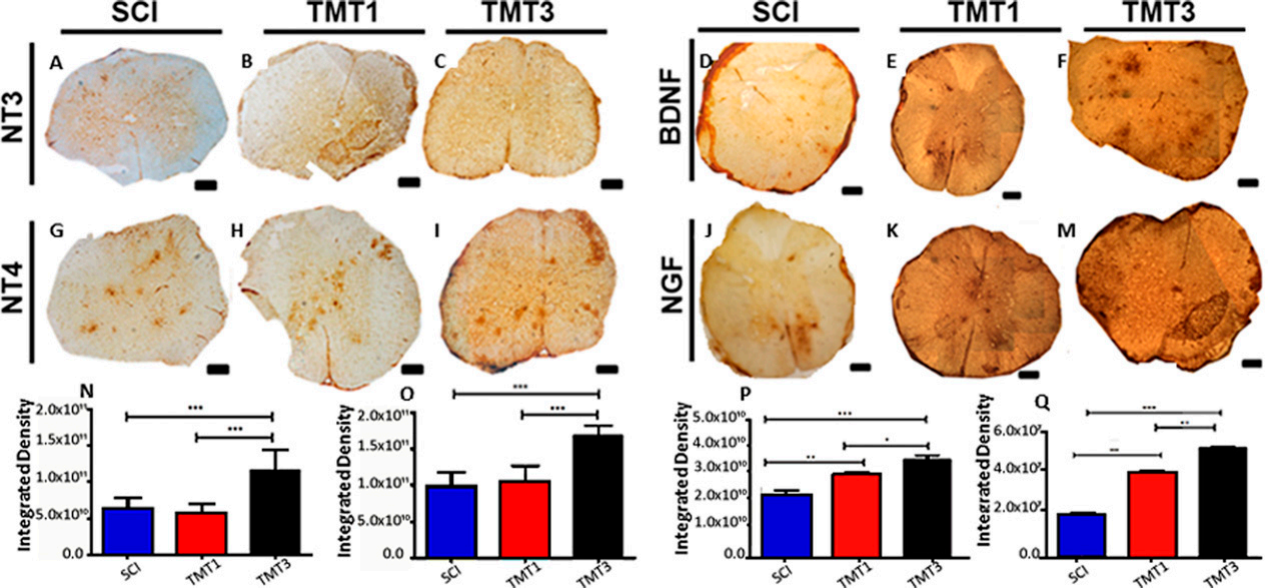
Exercise volume affected spinal cord neurotrophin expression. Exercised groups showed immunostaining for NT-3 **(A–C)**, BDNF **(D–F)**, NT-4 **(G–I)**, and NGF **(J–M)** as measured by integrated density analysis (N, O, P, and Q, respectively). Immunostaining was significantly more intense in TMT3 than in SCI and TMT1 for all neurotrophins analyzed. In addition, immunostaining intensity was also significant in TMT1 than in SCI for BDNF and NGF. Data were analyzed using one-way ANOVA and Tukey’s post test. ***p < 0.001, **p < 0.01, and *p < 0.05. Scale bar = 50 μm. BDNF, brain-derived neurotrophic factor; HGF, nerve growth factor; NT, Neurotrophin; SCI, spinal cord injury.

Eight weeks after injury, spinal cord tissue from all groups showed a diffuse immunoreactivity for NT-3, BDNF, NT-4, and NGF predominantly in the gray matter (Fig. 6). The immunostained area was significantly larger in TMT3 than in TMT1 and SCI for NT-3 (*p* < 0.001, both), NT-4 (*p* < 0.001, both), BDNF (*p* < 0.05 and *p* < 0.001, respectively), and NGF (*p* < 0.01 and *p* < 0.001, respectively). In addition, TMT1 also showed significant immunostaining compared with SCI for BDNF and NGF (*p* < 0.01 and *p* < 0.001, respectively).

**FIG. 7.**
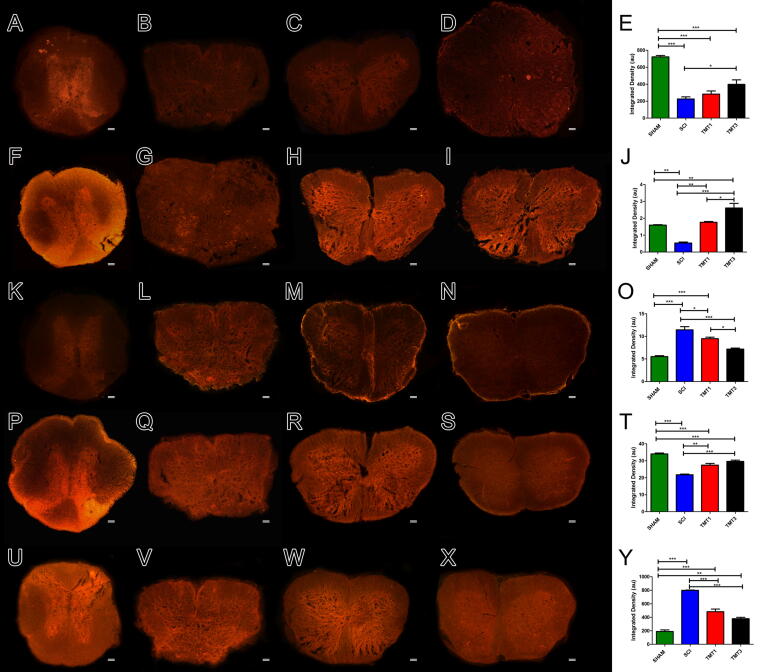
Exercise volume affected neuroplasticity, glial scarring formation, and neuroinflammation. Spinal cord tissue from all groups was analyzed for the expression of CC1 **(A–E)**, GAP-43 **(F–J)**, GFAP **(K–O)**, NF200 **(P–T)**, and TMEM 119 **(U–Y)** by integrated density analysis. Immunostaining intensity was significantly larger in TMT3 than in SCI for CC1 (*p* < 0.05, Fig. 7E), GAP-43 (*p* < 0.001, Fig. 7J), and NF200 (*p* < 0.001, Fig.7T), and significantly lower than SCI in relation to GFAP and TMEM119 (*p* < 0.001 for both, Fig. 7O and 7Y). TMT3 also showed a significant immunostaining difference compared with TMT1 for GAP-43 (*p* < 0.05). TMT1 showed significant difference to SCI for GAP-43 (*p* < 0.01), GFAP (*p* < 0.05), NF200 (*p* < 0.01), and TMEM119 (*p* < 0.001). Data were analyzed using one-way ANOVA and Tukey’s post-test. ***p < 0.001, **p < 0.01, and *p < 0.05. Scale bar = 50 μm. CC1, adenomatous polyposis coli clone; GAP-43, growth-associated protein-43; GFAP, glial fibrillary acidic protein; NF200, neurofilament protein 200; SCI, spinal cord injury; TMEM 119, transmembrane protein 119.

Expression of CC1, GAP-43, GFAP, NF200, and TMEM119 was analyzed by immunofluorescence eight weeks postinjury (Fig. 7). The immunostained area was significantly larger in TMT3 than in SCI for CC1 (*p* < 0.05, Fig. 7E), GAP-43 (*p* < 0.001, Fig. 7J), and NF200 (*p* < 0.001, Fig.7T), and significantly lower than SCI in relation to GFAP and TMEM119 (*p* < 0.001 for both, Fig. 7O and Y). These results suggest that TMT3 exhibits a pattern more suitable to myelination (CC1 positive) and nerve regeneration (GAP-43 and NF-200) than the inflammatory and hyper-reactive pattern (GFAP and TMEM119 less immunostained). TMT3 also showed a significant immunostaining difference compared with TMT1 for GAP-43 (*p* < 0.05), confirming the potential for regeneration in this TMT3 protocol. In addition, there were statistically significant differences in immunostaining intensity between TMT1 and SCI for GAP-43 (*p* < 0.01), GFAP (*p* < 0.05), NF200 (*p* < 0.01), and TMEM119 (*p* < 0.001). Lastly, the SCI group exhibited significantly more staining to GFAP (*p* < 0.05 and *p* < 0.001, respectively) and TMEM119 (*p* < 0.001 for both groups) when compared with the exercised groups (TMT1 *p* < 0.05 and *p* < 0.001, respectively; and TMT3 *p* < 0.001, for both antibodies).

## Discussion

Although several promising pharmacological therapies have reached the clinical trial stage, physical therapy is still the only established reality for rehabilitation after SCI.^17^ However, there is no consensus in the literature about the type and volume of exercise that is most beneficial for rehabilitation, as well as the optimal timing of exercise interventions after injury. The literature describes a wide range of exercise protocols using different intensities, volumes, frequencies, and types of exercise, reporting both positive and negative effects.^31,37–42^ Since multiple variables can influence the exercise outcomes after an injury, more investigation is necessary with controlled variables to know the ideal time to start the exercise after injury or even when changing the volume or intensity of exercise protocol used to obtain better regeneration and functional recovery. This case is more complicated in central nervous system injury with primary and secondary physiopathological events that can worsen the condition and influence the therapeutic outcomes. The SCI has a classification according to the time after injury as the acute, subacute, and chronic phase.^12^ These phases establish different therapeutic challenges and can guide the therapeutic needs.

Our results showed that mice from the TMT3 group showed better results, reaching the intermediate phase of recovery exhibiting plantar placing and the development of stepping, as described by Basso and collaborators (2006), and also had significant scores compared with TMT1 mice from day 42 postinjury and from 21 days after SCI, both maintained until the end of the trial. Basso and coworkers (2006) ranked the behaviors displayed by mice during locomotor recovery to reflect progression and categorized them into 3 phases. The early phase of recovery is related to paralysis or paresis, and ankle motion determines the end of this phase. The intermediate phase requires plantar placing, weight support, and the development of stepping, and the late phase highlights the fine details of locomotion, such as forelimb–hindlimb coordination, paw position during stance, and the extent of trunk stability. The SCI group just reached the initial phase of recovery, while the trained group developed until the intermediate phase, showing the impact of this training on recovery. Besides this, the outcomes were better when the volume of the exercise was related to the time after injury (TMT3) than compared with the unique volume protocol (TMT1) during all eight weeks, indicating that the adjustment to exercise volume to time after injury is relevant to be considered during rehabilitation.

TMT3 mice also showed significantly better results on the rotarod and LWT (for both paws) at 42 days postinjury. These TMT3 mice also showed significantly better results on the rotarod and LWT (for both paws) at 42 days postinjury. These results agree with previous studies that reported significant functional gains in mice that underwent treadmill training after SCI when assessed weeks after injury, especially in the chronic phase.^30,43^ Rotarod and LWT can be used as resources to assess the fine details of locomotion, such as forelimb–hindlimb coordination, paw position during stance, and the extent of trunk stability seen in the late phase of locomotor recovery.

The regaining of some sensitivity, voluntary movements, and the standing position after treadmill training may also be seen in human patients that showed clinical improvement on the ASIA (American Spinal Injury Association) impairment scale.^14,16,24^ TMT3 mice also showed better motor performance and exhibited a sensitivity threshold closer to that of healthy animals, suggesting that this exercise protocol could be a promising nonpharmacological therapeutic approach for rehabilitation of sensitive neural disorders.

Activity-based restorative strategies have been used in an attempt to restore and preserve muscle mass and some muscle functions after SCI, as well as to stimulate nerve regeneration and plasticity^44^; and the results of our trained groups are in agreement with these expected results. It has been reported that exercise can enhance motor control by activating residual neural circuitry, inducing axonal plasticity, and the formation and potentiation of synapses.^37,45^ The apparent plasticity of the preserved nerve fiber collaterals induces a greater activity of the CPG and allows for better locomotor performance during exercise, since it promotes the activation and modulation of the CPG through endogenous neuromodulators, such as serotonin, dopamine, and nitric oxide.^30,37,46–48^ Accordingly, the exercised groups in our study showed improvement in CMAP amplitude, as well as increased white matter preservation and myelinated nerve fiber count in the lesion area compared with the untreated group (SCI). This increased tissue preservation may account for adequate nerve conduction and greater muscle recruitment reflected in the CMAP amplitude. These results suggest that the benefits of exercise may be related to structural preservation and optimization of the conduction and propagation of action potentials and muscle recruitment. Nevertheless, the benefits of exercise after SCI need to be better understood because motor deficits still persist even after intensive, volume, and frequent rehabilitation.^26^

Skeletal muscle fibers can exhibit structural and functional adaptive changes after damage or training, which may ultimately lead to changes in fiber size (atrophy or hypertrophy) and fiber type (slow–fast or glycolytic-oxidative transitions) that represent the basis of plastic adaptation of muscle. For example, changes in fiber size reflect an imbalance in protein turnover with protein loss, leading to muscle atrophy.^49^ The exercise protocols in this study were adequate to prevent soleus and gastrocnemius muscle atrophy; thus, prevention of muscle atrophy alone would be sufficient to justify the therapeutic use of exercise after SCI. Exercised groups had significantly larger areas and weight from the soleus compared with SCI mice. One probable explanation for the muscle mass preservation is that exercise promotes a positive effect on neuromuscular plasticity, triggering increased muscle activity in the exercised animals.^50,51^ This weight difference between exercised groups and untrained animals was not evident for the gastrocnemius muscle, probably due to the type of muscle fiber recruited by the exercise performed in our study. Treadmill exercise recruits priority type I slow fibers, which are predominant in the soleus, whereas the gastrocnemius has a predominance of type II fast fibers^33^, as seen in the SHAM group.

Although there was no significant difference in gastrocnemius weight between the injured groups after exercise, we detected a change in the frequency distribution of type I and type II fibers among the exercised groups. The gastrocnemius muscle from TMT3 mice exhibited fiber-type plasticity, with fewer type II fibers and a predominance of type I fibers than that from SCI, TMT1, and SHAM mice. Fiber-type plasticity, which reflects variations in muscle structure, functional properties, and specialization, such as force or fatigue resistance, can be markedly modulated by training and detraining.^49,52,53^ This fiber-type plasticity stimulated by exercise can reflect a reprogramming of gene transcription and remodeling of fiber contractile properties (slow-fast transitions) or metabolic profile (glycolytic-oxidative transitions).^49,54^ The fiber-type composition of the muscle determines its functional properties and the ability to adapt to new functional demands.^39,53^ The highly specialized skeletal muscle fibers are selectively recruited and allow the execution of functional tasks. The fiber-type plasticity observed in the gastrocnemius of TMT3 is an important finding of our study because postural muscles, which are essential for locomotor function and posture, especially in bipedal posture, are primarily formed by type I fibers.^55^

We also observed that NT-3, BDNF, NT-4, and NGF levels increased in the spinal cord tissue of trained mice compared with untrained animals. This increased expression was mainly seen in TMT3 animals, suggesting that these neurotrophins can be modulated by exercise volume or by the time after the injury was performed the training. Different types and volumes of exercise may affect the production and function of neurotrophins in the spinal cord and muscle tissue.^20^ However, the last two decades have taught us that several neurotrophins^20,51^ and myokines^44,56,57^ can modulate neuromuscular functions and the neuroregenerative response to injury.^20^ Neurotrophins regulate cell survival and metabolism, including cell signaling pathways, protein synthesis, and synaptic plasticity.^58,59^ Neuromuscular plasticity induced by training is associated, at the molecular level, with positive regulation of neurotrophic factors.^20,60^ Thus, it is reasonable to suggest that the functional recovery after SCI observed in our study may be related to the increased expression of these neurotrophic factors.

TMT3 showed higher expression of CC1, GAP-43, and NF200 and lowest immunoreactivity to GFAP and TMEM119, suggesting compromise with a regenerative and noninflammatory profile since the oligodendrocyte and neuron markers are abundantly expressed and astrocytes and microglial reactivity are reduced when compared with the SCI group. The increased expression of CC1 may reflect the early survival of oligodendrocyte progenitor cells from the neuroprotective exercise event, which then differentiate into mature oligodendrocytes.^61,62^ Several studies have shown upregulated expression of GAP-43 and NF200 after SCI and treadmill training, indicating these proteins have a high potential to promote reinnervation and nerve sprouting from a lesion area, because they are associated with the sprouting and the presence of mature axons during neural regeneration and neuroplasticity.^63–65^ After SCI, high-volume exercise seems to favor protection against reduction of glial scar formation and inflammation from reactive microglia and astrocytes,^66,67^ as seen in our study by the reducing levels of GFAP and TMEM119. Thus, these results confirm that the TMT3 exercise protocol could promote neuroplasticity, neuroprotection, and inflammatory modulation and, consequently, regeneration and functional rehabilitation after injury.^66,68^

Despite this work clarifying some relevant points about the positive results from exercise as a therapeutic strategy after spinal cord injury, aiming neuromuscular plasticity and functional recovery, additional research must be necessary to respond to parameters for the physiological response related to exercise, and the mechanism related to the observed results.

## Conclusion

In conclusion, this work demonstrated that exercise volume can positively modulate neuromuscular plasticity, neuroregenerative response, functional recovery, and microenvironment modulation after SCI, and the results are correlated and can be improved by adjusting exercise volume according to the clinical phase and time after the injury. Therefore, this intervention seems promising to improve the regenerative process after central nervous system injury and can be easily translated to the clinical setting.

## Abbreviations Used

BDNF: brain-derived neurotrophic factor
BMS: Basso mouse scale
CMAP: compound muscle action potential
DAB: 3,3′-diaminobenzidine
ENMG: Electroneuromyography
IPI: immediately postinjury group
LWT: ladder walking test
NGF: nerve growth factor
NT-3: compound muscle action potential
NT-4: immediately postinjury group
PWT: paw withdrawal threshold
SCI: spinal cord injury
